# Supplementary material for: Comparative Study of Lycopene-Loaded Niosomes Prepared by Microfluidic and Thin-Film Hydration Techniques for UVB Protection and Anti-Hyperpigmentation Activity
Source: Int J Mol Sci. 2024 Oct 31;25(21):11717. doi: 10.3390/ijms252111717 (PMC11547095; doi:10.3390/ijms252111717)
Supplement: Supplementary file 1 [file ijms-25-11717-s001.zip › ijms-3249946-supplementary.pdf]

Supplementary Material

Supplementary Figures and Tables

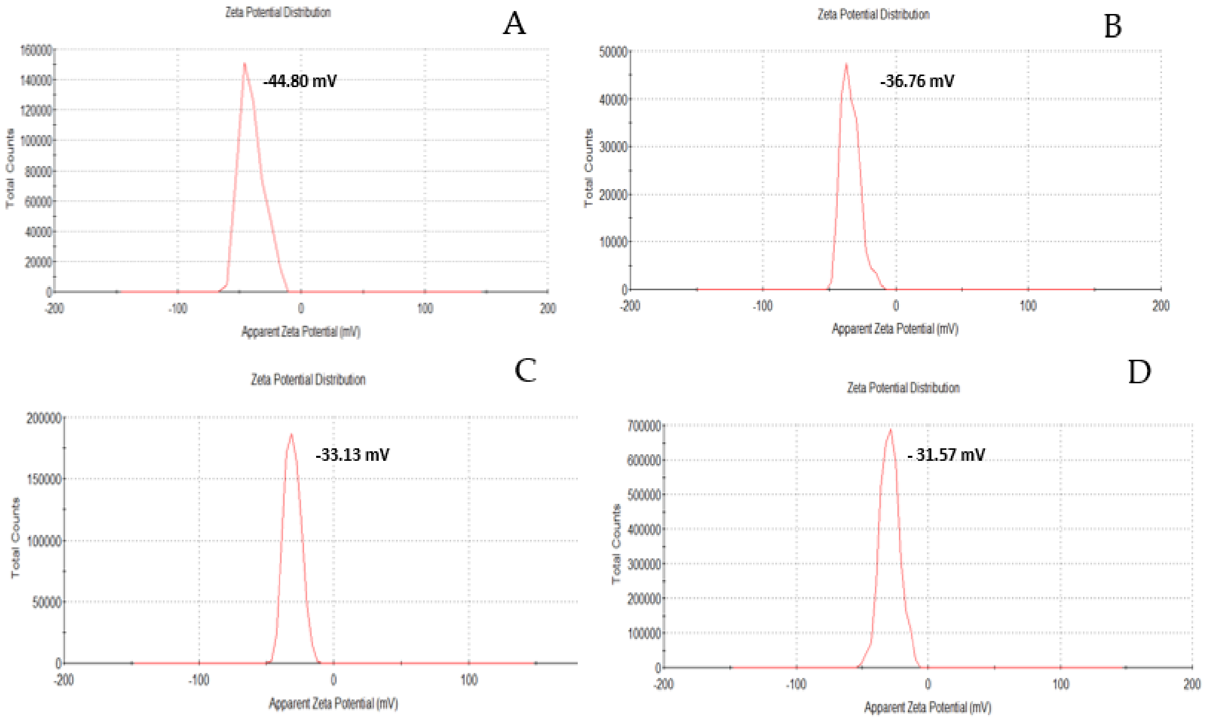

Figure S1. The zeta-potential graph of (A) BN–T, (B)TN–T, (C) CN–T, and (D) MRN–T

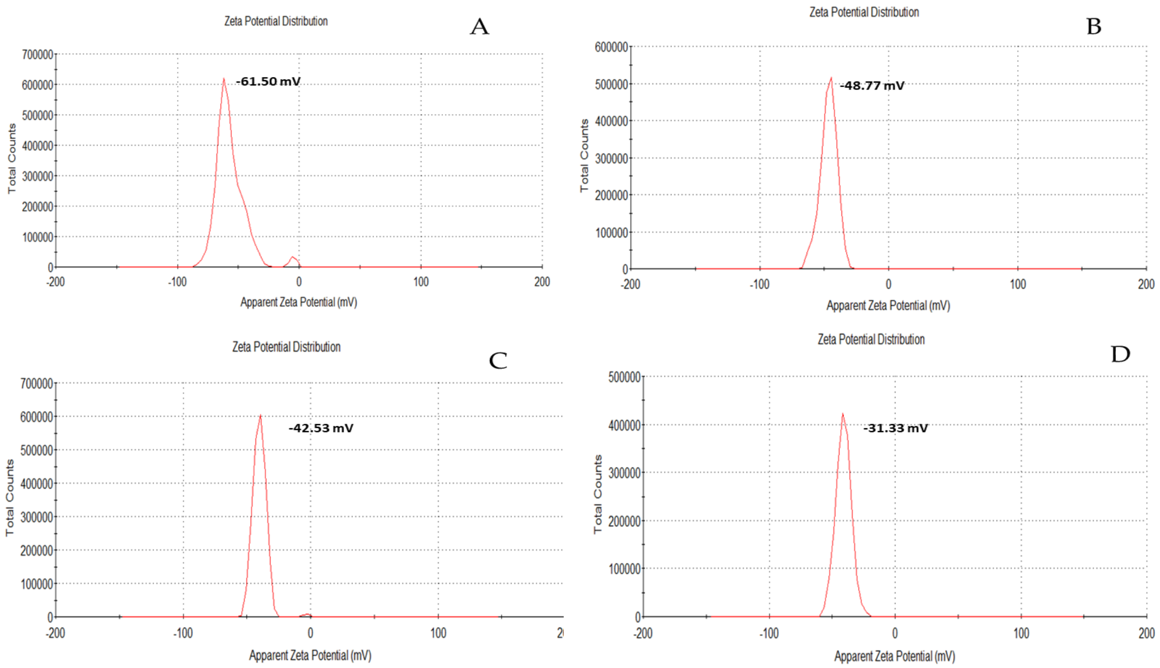

Figure S2. The zeta-potential graph of (A) BN–M, (B)TN–M, (C) CN–M, and (D) MRN–M

Table S1 Cumulative release of lycopene from tomato, carrot and mixed red vegetable and their niosome formulations from MF and TFH methods.

% Cumulative release of lycopene				SD		
**Time**	**TS**	**TN–M**	**TN–T**	**TS**	**TN–M**	**TN–T**
0.5	0.00	13.23	19.84	2.06	1.91	4.58
1	42.80	36.06	32.26	5.89	8.72	4.16
2	61.99	46.84	45.15	18.97	4.13	5.62
4	64.30	53.96	58.22	23.93	4.28	2.19
6	65.65	61.93	67.18	1.13	4.67	8.83
8	69.88	63.29	66.90	22.13	10.20	6.51
12	69.73	69.77	71.13	10.44	0.32	9.14
24	76.30	81.75	76.08	15.84	30.65	3.53
**% Cumulative release of lycopene**				**SD**		
**Time**	**CS**	**CN–M**	**CN–T**	**CS**	**CN–M**	**CN–T**
0.5	63.49	17.43	20.91	6.66	9.06	6.04
1	70.14	31.83	29.13	17.65	11.10	11.87
2	78.91	52.07	41.61	0.65	18.67	8.68
4	90.65	63.27	57.42	19.97	14.94	32.36
6	94.70	69.63	63.00	23.74	8.60	3.58
8	100.85	73.46	73.01	12.97	3.73	5.97
12	106.14	77.86	76.45	6.88	7.49	17.63
24	122.09	77.98	76.96	21.45	13.29	29.08
**% Cumulative release of lycopene**				**SD**		
**Time**	**MRS**	**MRN–M**	**MRN–T**	**MRS**	**MRN–M**	**MRN–T**
0.5	82.76	19.64	14.87	11.33	6.81	3.43
1	85.76	31.71	22.80	5.66	6.27	13.20
2	88.34	42.24	29.48	13.10	11.25	17.44
4	93.32	52.66	46.97	5.13	7.76	19.43
6	98.74	62.73	55.87	2.89	6.91	32.95
8	100.88	63.48	57.02	2.04	13.74	32.21
12	102.41	64.68	58.91	7.78	16.12	35.46
24	101.47	65.75	62.97	5.13	8.24	26.67

*TS = Tomato extract solution, CS = Carrot extract solution (CS), MRS = Mixed red vegetable solution, Niosome from MF method ; TN–M = niosome entrapped tomato extract, CN–M = niosome entrapped carrot extract, MRN-M = niosome entrapped mixed red vegetable
Niosome from TFH method ; TN–T = niosome entrapped tomato extract, CN–T = niosome entrapped carrot extract, MRN–T = niosome entrapped mixed red vegetable

Table S2. Kinetic model prediction of lycopene from tomato, carrot, and red vegetable extracts and BN–T, TN–T, CN–T and MRN–T, respectively. The formulations prepared by MF method are BN–M, TN–M, CN–M and MRN–M, respectively.

Sample	Zero order		First order		Higuchi		Korsmeyer-Peppas		
	R^2	K_0	R^2	K_1	R^2	K_H	R^2	n	Kp
TS	0.5545	0.9958	0.4724	0.0070	0.7071	6.7594	0.7963	0.1549	5.4260
TN– M	0.8370	1.7380	0.7320	0.0127	0.9537	11.1530	0.9848	0.2498	4.8477
TN–T	0.6020	1.5345	0.5161	0.0121	0.7885	10.5540	0.8980	0.2699	4.7812
CS	0.8728	2.0348	0.8070	0.0091	0.9734	12.9170	0.9969	0.1718	6.3389
CN–M	0.5073	1.5067	0.4264	0.0115	0.7062	10.6850	0.8301	0.2726	4.9096
CN–T	0.5828	1.7734	0.5070	0.0142	0.7777	12.3140	0.8997	0.3214	4.5823
MRS	0.5424	0.6267	0.5353	0.0029	0.7392	4.3975	0.8931	0.0630	6.9254
MRN–M	0.4985	1.1793	0.4533	0.0102	0.7000	8.3999	0.8615	0.2395	4.7242
MRN–T	0.5666	1.4840	0.4924	0.0151	0.7573	10.3130	0.8803	0.3436	4.0776

Lycopene from tomato, carrot, and red extracts and niosome formulations (The formulations prepared by MF method are BN-M, TN–M, CN–M and MRN–M, and the formulations prepared by TFH method are BN–T, TN–T, CN–T and MRN–T. K_0 is a constant of zero-order, K_1 is a constant of the first-order, K_H is a constant of the Higuchi model, K_P is a constant of the Korsmeyer–Peppas model and n is the diffusional exponent characteristic of release from the Korsmeyer–Peppas model.

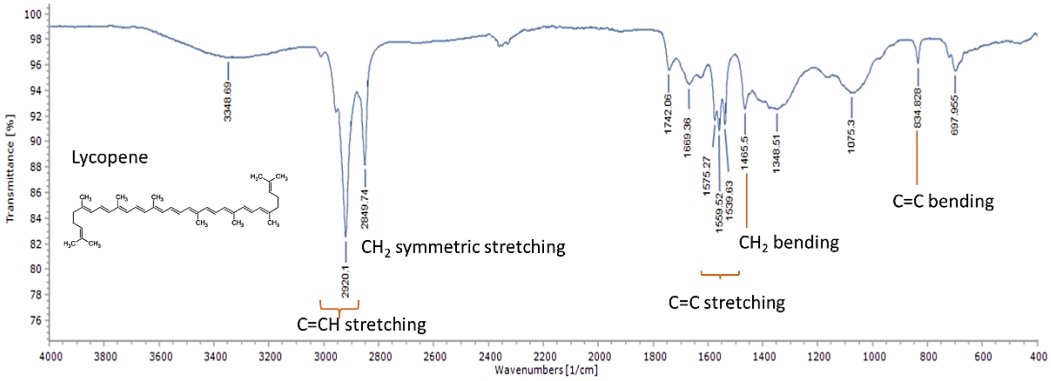

(A)Wavenumbers of Lycopene (cm^{-1})	Functional groups
2920.10	C=CH stretching
2849.74	CH_2 symetric stretching
1539.63, 1559.52, 1575.27	C=C stretching
1465.50	CH_2 bending
1075.30	C–C stretching
834.83	C=C bending

Figure S3 FTIR spectrum of Lycopene

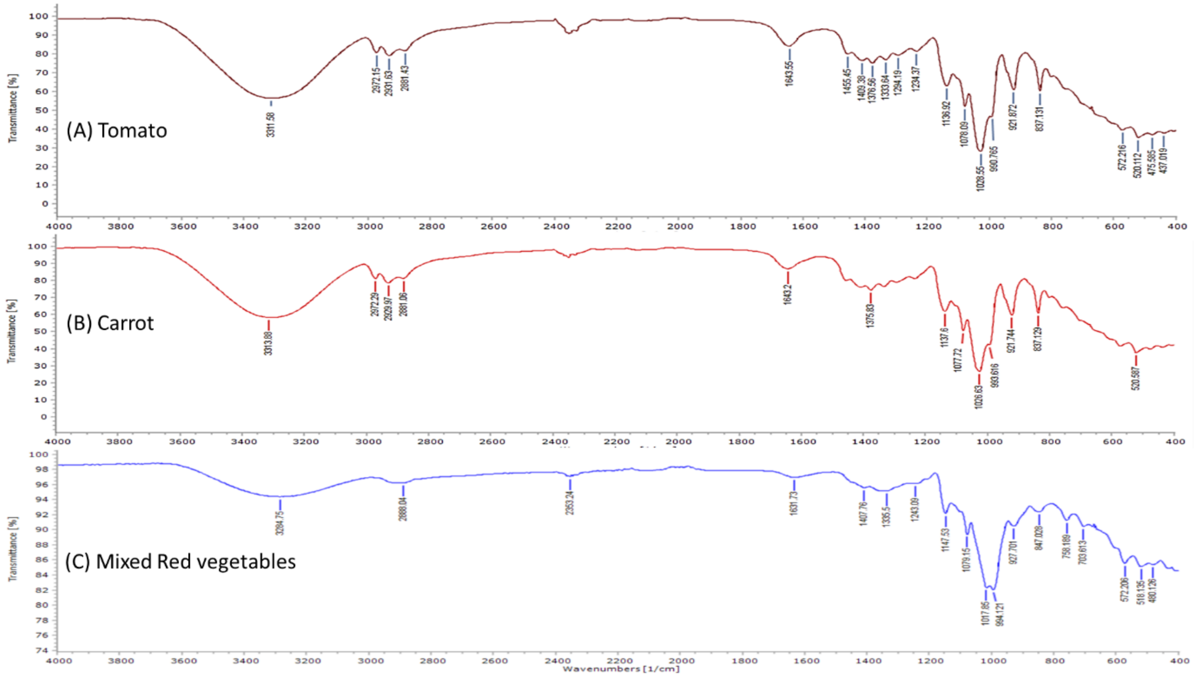

Figure S4 FTIR spectra of Tomato (A), Carrot (B) and Mixed red vegetables (C)

Wavenumbers (cm^{-1})			Functional groups
Tomato	Carrot	Mixed red vegetables	
3311.58	3313.88	3284.75	O–H stretching
2972.15<br>2931.63<br>2881.43	2972.29<br>2929.97<br>2881.06	2888.04	C–H stretching
1376.56	1375.83	1335.50	CH_3 bending
1028.55	1026.63	1016.42	C–C stretching

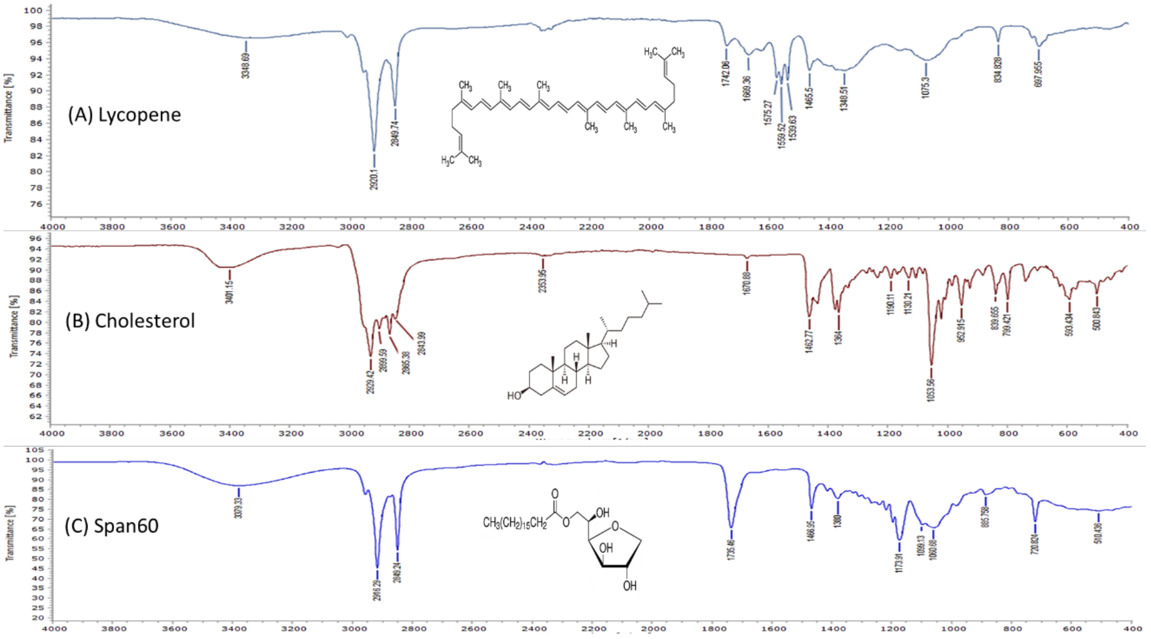

Figure S5 FTIR spectra of Lycopene (A), Cholesterol (B) and Span 60 (C)

(A)Wavenumbers of Lycopene (cm^{-1})	Functional groups
2920.10	C=CH stretching
2849.74, 2849.74	CH_2 symetric stretching
1559.52, 1539.63, 1669.36	C=C stretching
1465.50	CH_2 bending
834.83	C=C bending
(B) Wavenumbers of Cholesterol (cm^{-1})	Functional groups
3401.15	O-H stretching (–OH)
2929.42, 2899.59, 2865.38, 2843.99	C–H stretching
1670.88	C=C stretching
1462.77	CH_2 bending
1053.56	C–O stretching of alcohol
(C) Wavenumbers of Span 60 (cm^{-1})	Functional groups
3379.33	O–H stretching
2916.29, 2849.24	Strong C–H stretching
1735.46	C=O strong ester bonding
1466.95	CH_2 bending
1173.91	C–O stretching
720.82	CH_2 Rocking

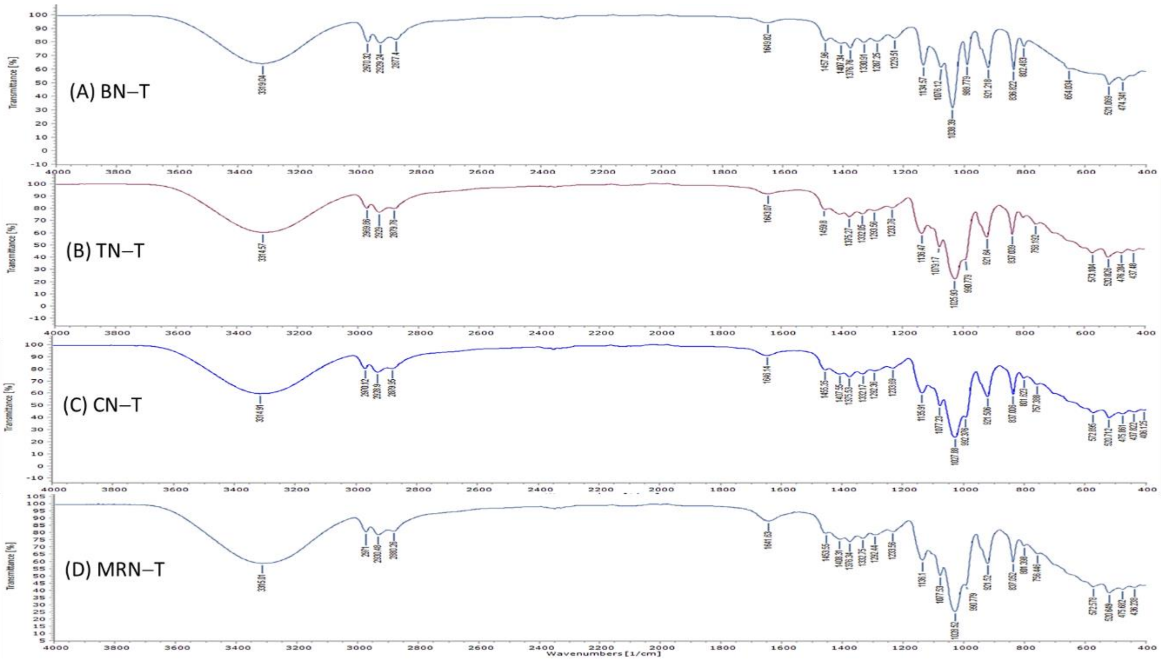

Figure S6 FTIR spectra of niosome prepared by thin film hydration method: BN–T (A,), TN–T (B), CN–T (C) and MRN–T (D) (BN–T = Blank niosome, TN–T = Tomato niosome, CN–T = Carrot niosome and MRN–T = Mixed red vegetables niosome)

Wavenumbers (cm^{-1})				Functional groups
BN–T	TN–T	CN–T	MRN–T	
3319.04	3314.57	3314.91	3315.99	O–H stretching
2970.32<br>2929.24<br>2877.40	2969.86<br>2929.00<br>2879.76	2970.12<br>2928.90<br>2879.95	2970.38<br>2930.39<br>2878.13	C–H stretching
1649.82	1643.07	1646.14	1649.18	C=C stretching
1457.96	1454.92	1455.36	1457.51	CH_2 bending
1038.39	1025.93	1027.88	1037.18	C–O stretching
989.78	990.78	992.38	989.90	=C–H out of plane bending

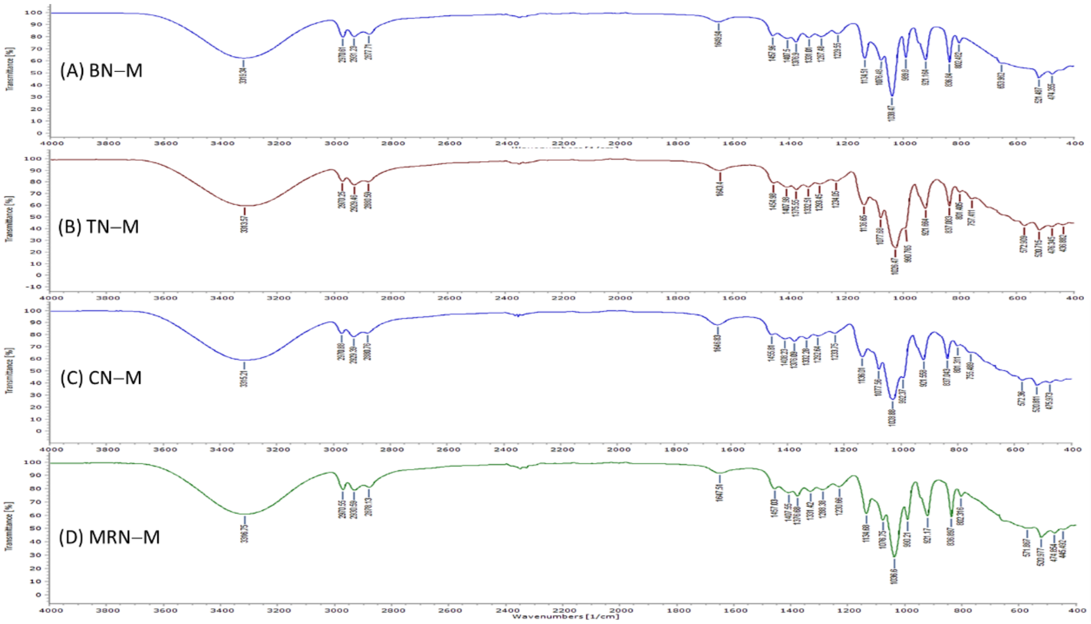

Figure S7 FTIR spectra of niosome prepared by microfluidic method: BN–M (A), TN–M (B), CN–M (C) and MRN–M (D) (BN–M = Blank niosome, TN–M = Tomato niosome, CN–M = Carrot niosome and MRN–M = Mixed red vegetables niosome)

Wavenumbers (cm^{-1})				Functional groups
BN–M	TN–M	CN–M	MRN–M	
3319.34	3313.57	3315.21	3316.75	O–H stretching
2970.61<br>2931.23<br>2877.71	2970.25<br>2929.46<br>2880.59	2970.88<br>2929.39<br>2880.76	2970.55<br>2930.59<br>2878.13	C–H stretching
1649.94	1643.40	1646.83	1647.51	C=C stretching
1457.96	1454.98	1455.36	1457.03	CH_2 bending
1038.47	1026.47	1028.88	1036.60	C–O stretching
989.80	990.76	992.37	990.21	=C–H out of plane bending

The slight shift from the typical cholesterol peak (960–980 cm^{-1}) to 989 cm^{-1} might be due to the interactions between Span 60 and cholesterol in the niosome structure.

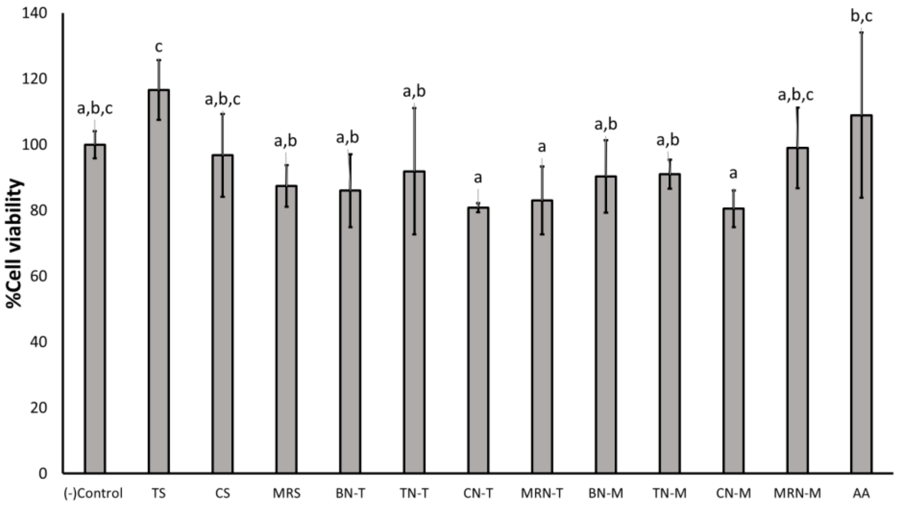

Figure S8. Cell viability of HaCaT cells treated for 24 h by tomato (TS), carrot (CS), mixed red vegetable extracts (MRS) and niosome formulations (BN–T, TN–T, BN–M, TN–T, CN–T, MRN–T, BN–M, TN–M, CN–M, MRN–M and 50 µg/mL ascorbic acid (AA). Data represent the mean ± SD values of the three replicates. Significance was evaluated by Duncan's test (p<0.05). a,b,c letters indicate significant differences between the group.

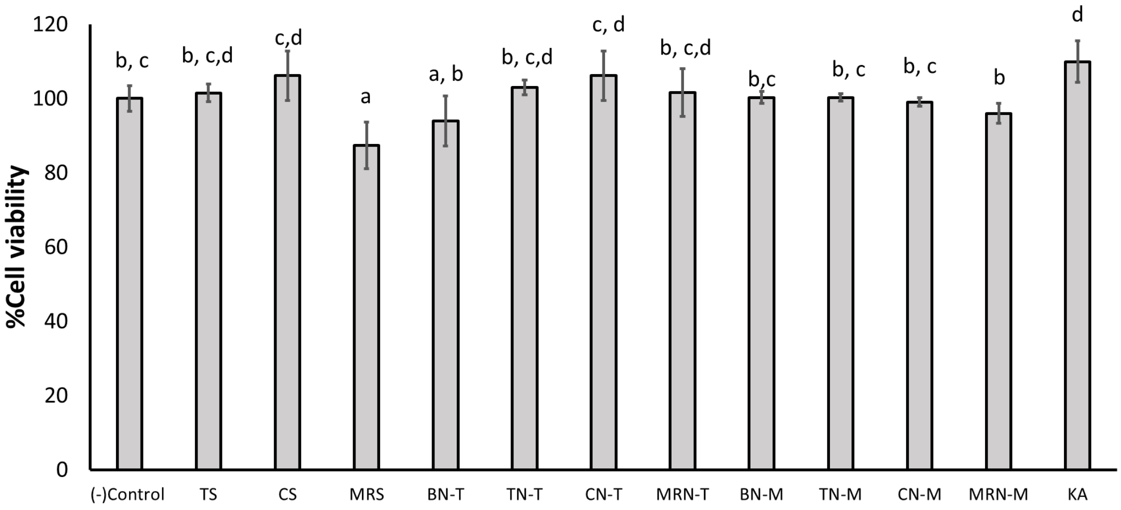

Figure S9. Cell viability of B16F10 cells treated for 24 h by tomato (TS), carrot,(CS), mixed red vegetable extracts (MRS), the niosome formulations (BN–T, TN–T, CN–T, MRN–T, BN–M, TN–M, CN–M, MRN–M) and 100µg/mL kojic acid (KA). Data represent the mean ± SD values of the three replicates. Significance was evaluated by Duncan's test (p<0.05). a,b,c,d letters indicate significant differences between the group.

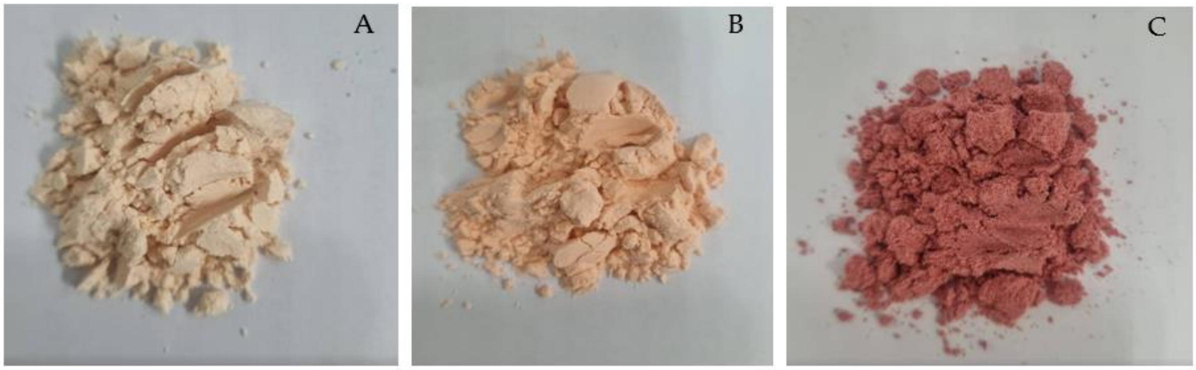

Figure S10. Lycopene extract powders from (A): tomato (T), (B): carrot (CS) and (C): mixed red vegetables (MR)

HPLC method validation for Lycopene determination.

HPLC analysis for confirmation of lycopene in vegetables (Sathish et al. 2009). The HPLC method validation for lycopene is as follows.

1) An HPLC system (2000 series; Agilent Technologies, Inc., Palo Alto, CA, USA) coupled with a C18 column (250 × 4.6 mm, 5 μm; Phenomenex., USA) and UV-VIS detector at a wavelength of 475 nm was used in this study. The column temperature was 25 ◦C, and the injection volume was 20 μL. The flow rate was 1 mL/min. The isocratic system for the mobile phase with acetonitrile, methanol, and tetrahydrofuran (70:25:5, % v/v). The chromatographic run was set for 20 min.

2) Method validation

- Linearity and Range: The lycopene standard solution was determined in concentration (0.13, 12.5, 25, 50, 100, 150, 200, 250 μg/mL) The different lycopene solutions were injected into the triplicated, and a calibration curve was constructed for linear regression. The correlation coefficient (R^2) of the regression equation was obtained to validate the linearity parameter

- Limit of Detection (LOD) and Limit of Quantification (LOQ)

LOD and LOQ were determined depending on the calibration curve and standard deviation of the response obtained through the analysis of 10 blank samples analyzed in the triplicate following equation: LOD = 3.3 × δ/s (1), LOQ = 10 × δ/s (2)

where "δ" represents the standard deviation of the response, and "s" represents the calibration curve slope.

-Precision

The precision parameter of the analysis method, intra-day, and inter-day variability were evaluated. The lycopene standard solution was used at concentrations in the range of 12.5 – 250 μg/mL. Validation was performed on the intra-day and inter-day variability in triplicate on the same day and on five different days.

-Accuracy

The accuracy parameter of the analysis method. The lycopene standard solution was used at three different concentrations in the range of 12.5 – 250 µg/mL. The percentage recovery values for lycopene were obtained from these results.

Results :

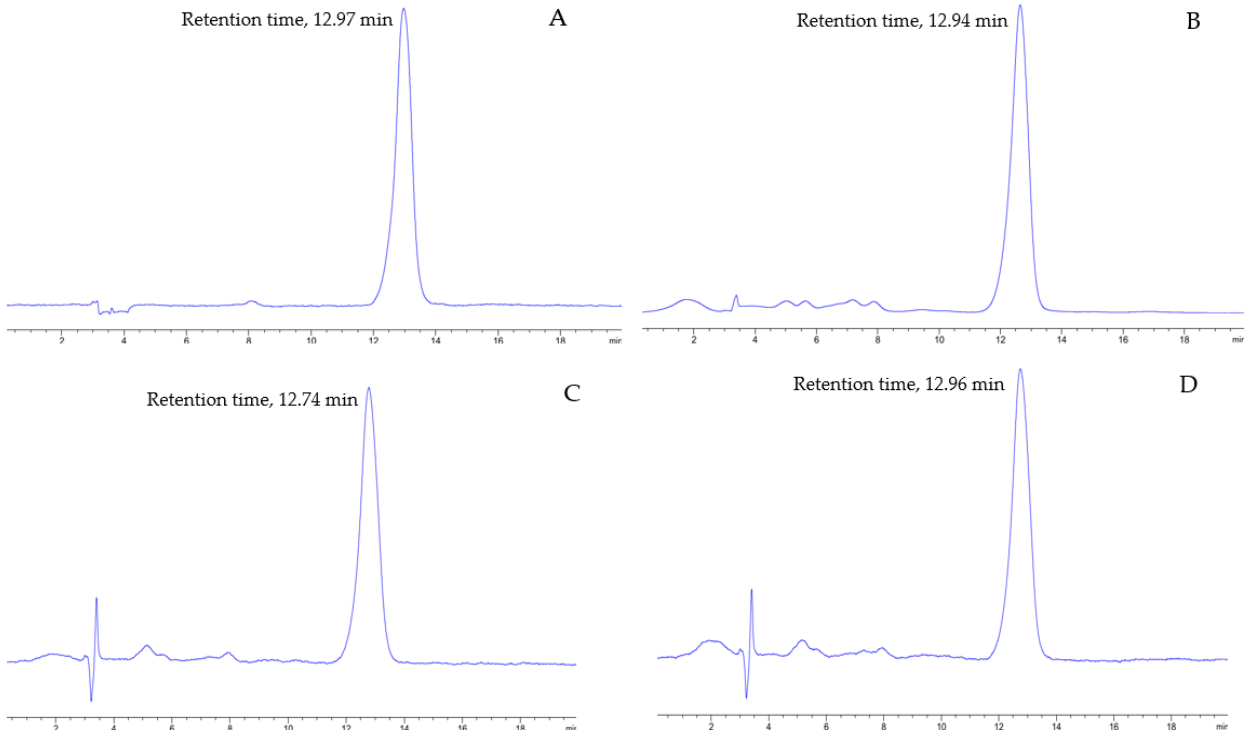

Figure S11 HPLC chromatogram: A; 250 µg/mL of lycopene standard, B; 10% of Extract powders from tomato (T), C; 10% of Extract powders from carrot (CS), D; 10% of extract powders from mixed red vegetables (MR)

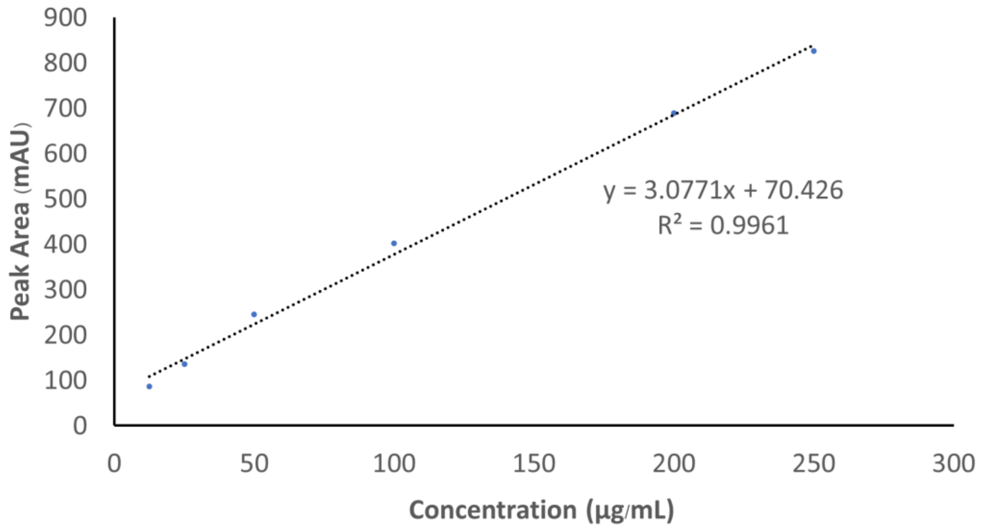

Figure S12 The calibration curve was constructed for a linear regression. The correlation coefficient (R^2) of the regression equation

Table S3 Results of linearity regression, correlation coefficient, LOD, LOQ, %RSD, and %Recovery of lycopene by HPLC

Parameter	Results
Linear	Y = 3.0771X – 70.426, R^2 = 0.996
LOD, LOQ (µg/mL)	LOD = 3.67, LOQ = 11.13
**Precision (n=5)**	
Conc (µg/mL) 25, 100, 250	
Within day (%RSD)	1.45 – 2.26%
Between day (%RSD)	0.18 – 2.79 %
**Accuracy (n = 5)**	
Conc (µg/mL) 30, 120, 240	
%Recovery	97.73 – 106.03%

Table S4 Results of lycopene content by UV-vis spectrophotometry and HPLC of Tomato, Carrot, and Mixed red vegetables

**Sample**	**Lycopene content (mg/100 g)**	
	**UV-vis spectrophotometry**	**HPLC**
Tomato (T)	165.65 ± 5.78	166.12 ± 0.60
Carrot (CS)	23.17 ± 0.60	23.05 ± 0.16
Mixed red vegetables (MR)	91.64 ± 25.65	91.78 ± 0.14

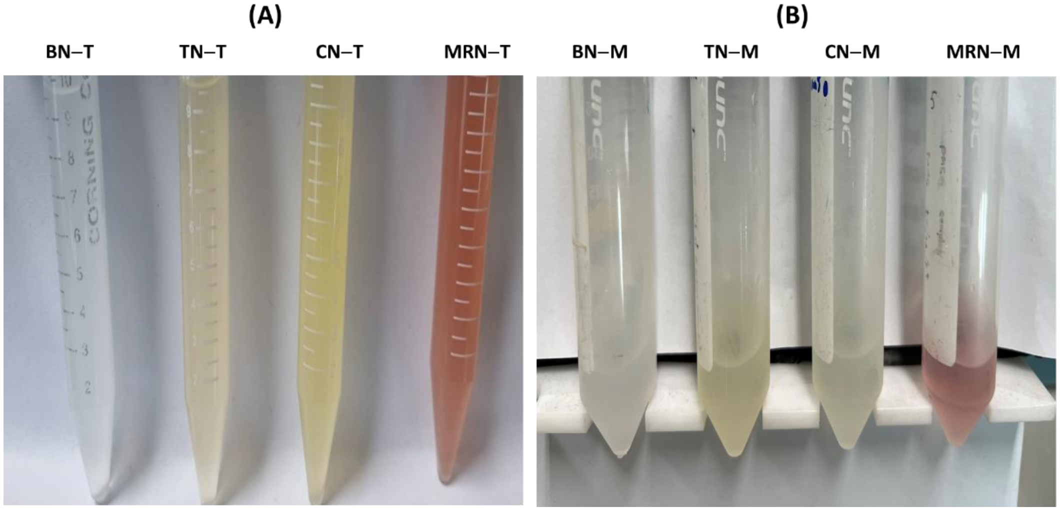

Figure S13 Characteristic of samples (A) Thin film hydration method; Blank Niosome (BN–T), Tomato Niosome (TN–T), Carrot Niosome (CN–T) and Mixed red vegetables niosome (MRN–T), (B) Microfluidic method; Blank Niosom (BN–M), Tomato Niosome (TN–M), Carrot Niosome (CN–M) and Mixed red vegetables niosome(MRN–M)
